# Exploring Human-Data Interaction in Clinical Decision-making Using Scenarios: Co-design Study

**DOI:** 10.2196/32456

**Published:** 2022-05-06

**Authors:** Helena Tendedez, Maria-Angela Ferrario, Roisin McNaney, Adrian Gradinar

**Affiliations:** 1 School of Computing and Communications Lancaster University Lancaster United Kingdom; 2 School of Electronics, Electrical Engineering and Computer Science Queen's University Belfast Belfast United Kingdom; 3 Department of Human Centred Computing Monash University Melbourne Australia; 4 Lancaster Institute for the Contemporary Arts Lancaster University Lancaster United Kingdom

**Keywords:** data-supported decision-making, health care professionals, respiratory care, scenario-based design, clinical decision-making, decision support, COPD, respiratory conditions, digital health, user-centered design, health technologies

## Abstract

**Background:**

When caring for patients with chronic conditions such as chronic obstructive pulmonary disease (COPD), health care professionals (HCPs) rely on multiple data sources to make decisions. Collating and visualizing these data, for example, on clinical dashboards, holds the potential to support timely and informed decision-making. Most studies on data-supported decision-making (DSDM) technologies for health care have focused on their technical feasibility or quantitative effectiveness. Although these studies are an important contribution to the literature, they do not further our limited understanding of how HCPs engage with these technologies and how they can be designed to support specific contexts of use. To advance our knowledge in this area, we must work with HCPs to explore this space and the real-world complexities of health care work and service structures.

**Objective:**

This study aimed to qualitatively explore how DSDM technologies could support HCPs in their decision-making regarding COPD care. We created a scenario-based research tool called Respire, which visualizes HCPs’ data needs about their patients with COPD and services. We used Respire with HCPs to uncover rich and nuanced findings about human-data interaction in this context, focusing on the real-world challenges that HCPs face when carrying out their work and making decisions.

**Methods:**

We engaged 9 respiratory HCPs from 2 collaborating health care organizations to design Respire. We then used Respire as a tool to investigate human-data interaction in the context of decision-making about COPD care. The study followed a co-design approach that had 3 stages and spanned 2 years. The first stage involved 5 workshops with HCPs to identify data interaction scenarios that would support their work. The second stage involved creating Respire, an interactive scenario-based web app that visualizes HCPs’ data needs, incorporating feedback from HCPs. The final stage involved 11 one-to-one sessions with HCPs to use Respire, focusing on how they envisaged that it could support their work and decisions about care.

**Results:**

We found that HCPs trust data differently depending on where it came from and who recorded it, sporadic and subjective data generated by patients have value but create challenges for decision-making, and HCPs require support in interpreting and responding to new data and its use cases.

**Conclusions:**

Our study uncovered important lessons for the design of DSDM technologies to support health care contexts. We show that although DSDM technologies have the potential to support patient care and health care delivery, important sociotechnical and human-data interaction challenges influence the design and deployment of these technologies. Exploring these considerations during the design process can ensure that DSDM technologies are designed with a holistic view of how decision-making and engagement with data occur in health care contexts.

## Introduction

### Background

Chronic obstructive pulmonary disease (COPD) is one of the most common chronic respiratory conditions in the world [1]. COPD typically arises from long-term exposure to airway irritants, such as cigarette smoke or air pollution [2-5]. It causes nonreversible chronic obstruction of the airways, resulting in breathlessness, fatigue, and frequent chest infections [6-8]. These symptoms can make it difficult to engage in daily activities, such as leaving home, socializing, and getting dressed [9,10]. Exposure to respiratory infections, physical exertion, smoke inhalation, and environmental factors such as air pollution can worsen symptoms [4,11-13]. COPD is a considerable challenge for millions of people and many health care services around the world [14,15]. It is estimated that ≥200 million people have COPD worldwide [16], with approximately 16 million people in the United States and 1.2 million in the United Kingdom [16,17]. In the United Kingdom specifically, COPD generates ≥140,000 hospital admissions annually, with 97% of these admissions being for emergency care [17,18].

When caring for patients with COPD, health care professionals (HCPs) must make timely and informed decisions to treat patients effectively. Clinical decision-making is a complex process that involves using medical knowledge to make decisions about care [19]. Making informed clinical decisions about patients with chronic conditions such as COPD requires quick access to a range of information about the patient and their medical history [14,20,21]. Insight into symptoms, quality of life, medications, past interventions, and results of recent clinical tests can add important context to inform decisions [21,22]. For example, by knowing the frequency and context of a patient’s respiratory exacerbations (ie, flare-ups of their COPD), HCPs can suggest more personalized interventions that may be more effective.

However, data relevant for chronic condition care are heterogeneous and often buried across paper notes or electronic records or held by other HCPs involved in the patient’s care [14,22-24]. Effectively collating and visualizing data about a patient’s chronic condition has the potential to support timely and informed care decisions [23-25]. This presents an opportunity to explore how digital technology can be designed to provide timely data-driven support for HCPs. Digital technology, which provides data that support decision-making, is termed data-supported decision-making (DSDM) technology. Designing DSDM technologies to support demanding health care contexts requires us to work closely with HCPs to explore their needs and expectations. An appreciation of the broader complexities of health care work is also needed [23].

In response, our research aimed to actively engage HCPs in considering how DSDM technologies could support clinical decision-making in the context of COPD care. Through extensive engagement with HCPs, we identified a set of data interaction scenarios relevant to their practice. We then created an interactive web application as a tool to visualize these scenarios and facilitate discussion about how DSDM technologies might support their work.

### DSDM Technologies in Health Care

Electronic health records [26], dashboards [27,28], and clinical decision support systems [29,30] are types of DSDM technologies used across health care. They present pertinent information to inform clinical decision-making. Dashboards are a prominent form of DSDM technology that can improve patient care [27,28,31-34]. Dashboards aggregate and visualize data in ways that produce insights to users. For instance, to support users to increase the number of patients undergoing health screening [31], identify possible high-risk medication prescribing scenarios [32], track in-patients in mental health wards [33], and effectively use patient-reported outcome data for cancer care [34].

While reviewing the literature on how DSDM technologies are designed and used in health care, we found that many studies focused on measuring the clinical effectiveness or quantified outcomes achieved using the technology [25,28,29,31,32,35,36]. Although these studies are crucial for establishing the quantitative impact of DSDM technologies on health care, they do not document the design process or provide detailed user insights about the technology. This knowledge is crucial to inform how DSDM technologies should be designed for real-world contexts from a human-centered perspective [37-40]. Collaborating with HCPs during the design process can unearth important sociotechnical and human-data interaction considerations required to build successful technologies [41-43]. Sociotechnical considerations investigate the design and implementation of systems based on technical and social dimensions [44]. Human-data interaction investigates how people interact with, interpret, and understand data [45,46]. Legibility, agency, and negotiability are key human-data interaction challenges [46]. Legibility refers to making data and algorithms transparent and comprehensible. Agency is the capacity to act on data and data implications. Negotiability relates to re-evaluating decisions about data and data processing as contexts change.

We found that a small number of studies have investigated the challenges involved in designing DSDM technologies for specific clinical contexts [47-49]. Bardram and Nørskov [48] and Sarcevic et al [47,49] took a user-centered approach to inform the design of context-aware dashboards for high-risk settings. Their prototypes focused on patient safety in operating theaters [48] and trauma resuscitation [47,49] and were evaluated with staff during a simulated clinical scenario. The researchers then revised how the data were presented to effectively support decision-making in these contexts, for instance, supporting dynamic information visualization in the fast-paced setting [48] and excluding audio feedback that could startle patients [49]. The nuances of clinical work were understood by engaging hospital staff during the design process, highlighting the value of partnering with end users when designing DSDM technologies [44,50]. However, there is more to learn about designing DSDM technologies outside the specific use case of high-risk settings [47-49]. Crucially, we need to explore wider everyday data interactions to inform the design of DSDM technologies in health care settings. This can enhance our understanding of the possibilities for DSDM technologies in health care.

### Scenario-Based Design in Health Care

Scenario-based design has been used in previous studies to evaluate health care technologies [43,48,49,51,52]. Scenarios, which are task-driven descriptions of work instances, focus on how a system can support human activities [51,53]. They are effective for the qualitative systematic evaluation of usability, suitability, and user experience of a technology or prototype [51,53]. This involves users completing tasks presented as scenarios on the proposed tool, presented as scenarios, and evaluating their experience.

Scenarios are particularly effective in eliciting detailed feedback from users without deploying a full system in clinical practice [52]. For example, Bardram [51] used scenarios to approach the redesign of an information system used in hospitals. Using scenarios allowed the hospitals’ existing activities to remain central to the design and evaluation, helping to focus on how the system could support both current and future activities. Scenarios are a creative thinking tool for envisaging how systems can support work and how it is organized [51]. Given our desire to capture rich details about how DSDM technologies could support HCPs with COPD care, scenario-based design was an appropriate method for this study.

### Study Aims

This study explored how DSDM technologies could support HCPs in their decision-making regarding COPD care. We achieved this by presenting an exploration of a scenario-based research tool called Respire. Respire is an interactive web app that presents HCPs with data interaction scenarios to support their decision-making about their patients with COPD and service. We designed Respire with input from 9 respiratory HCPs over 2 years and subsequently explored the output with 11 respiratory HCPs (9 of which were involved in the design process).

Our findings uncover the challenges faced when HCPs interact with health care data in context. From this, we reveal novel insights and lessons regarding the design of DSDM technologies to support the real-world complexities of clinical decision-making. Our paper makes three main contributions: (1) we provide insights into how DSDM technologies can support respiratory care by exploring HCPs’ data needs; (2) we uncover key barriers that impact HCPs’ engagement with data for decision-making; and (3) we provide novel and translatable [50] design implications that inform the creation of future DSDM technologies for health care.

### Study Structure

This study was divided into 3 stages. The first stage explores HCPs’ data needs related to COPD care, with a view to understanding how DSDM technology could support these requirements. The second stage involves the selection of key data requirements identified from the first stage and developing them into digital *data interaction scenarios* (presented in Respire). The third stage explores Respire with HCPs to understand how each data scenario could support their decision-making regarding COPD care.

## Methods

### Overview

This was a co-design study involving HCPs from 2 collaborating National Health Service organizations in North West England. Co-design involves embedding users in the design process, which is appropriate, given our desire to explore HCPs’ needs and experiences at each stage in detail [54]. The first organization we worked with, *the hospital*, has a respiratory ward with patients with COPD under the care of specialists. The specialists also visit respiratory outpatients in clinics, including those recently discharged from the hospital after an exacerbation. The second organization, *community care*, provides services that enable patients with COPD to manage their condition in the community. Services include routine clinics to assess a patient’s condition and management, pulmonary rehabilitation classes that use exercise and education to improve self-management [55], and home support services in which on-call specialized nurses support acute patients in their homes [56]. Patients are referred to community care by their general practitioner (GP) if they require advanced support or by the hospital to help stabilize their management after a hospitalization.

We have previously worked with HCPs from both organizations to explore their challenges with lack of access to data and effective visualizations for COPD care [24]. In a study by Tendedez et al [24], we found that (1) HCPs used multiple clinical systems that were inflexible, (2) existing data lacked required detail and quality, and (3) HCPs rarely had time for extensive training on clinical systems and needed intuitive user interfaces. Following Tendedez et al [24], we worked with them to explore how DSDM technology could support their needs by creating Respire. Respire is a web app designed to effectively visualize COPD data that are routinely collected across both organizations (contained in digital systems or paper notes). Crucially, it also aimed to visualize data that are not yet available in clinical practice to envision how decision-making could be supported in the future. The current and future data needs were identified through an iterative process.

We had no access to the organization’s data during this study. Therefore, Respire used test data sets that were created by the research team and based on the data requirements elicited during the study. A researcher experienced in large-scale hospital data advised on the content and structure of the test data sets. For instance, they advised on (1) the typical data fields that hospitals collect for patients with COPD and (2) the range of data within those fields. For scenarios that displayed medical data, we used web-based resources regarding medical readings to inform clinically realistic test values [57]. The researcher then checked that the test data sets we had produced were realistic for the purposes of the activity. The test data were created solely to populate Respire for this study; they were not intended to be used beyond this purpose. We stored the data in a MySQL database, which was read by Respire via a custom REST (Representational State Transfer) application programming interface.

**Figure 1:**
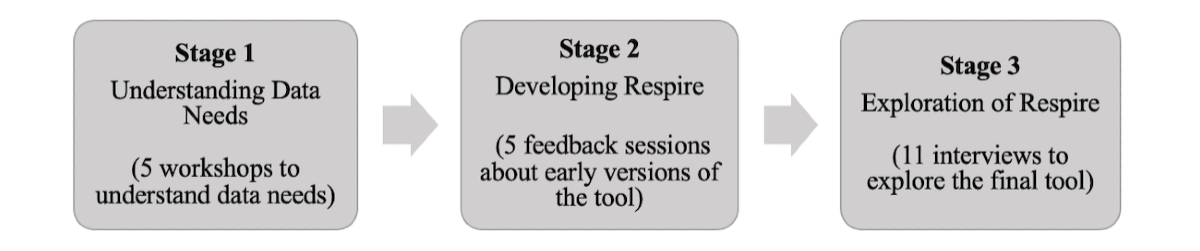
The 3 stages of the study methods.

An outline of the study methodology is presented in Figure 1.

### Participants

This study involved 11 HCPs across both organizations (6 from community care). Each participant (except for C6 and H5) was involved in the design process. Snowball sampling was used to recruit participants, with HCPs informing their colleagues about the project [58]. Details of the participants is presented in Table 1, showing their experience in their current role and using clinical information systems.

**Table 1:** Details of study participants.

Participant identifier^a^	Role	Years in current role	Experience using clinical information systems (years)
H1	COPD^b^ nurse	<1	3
H2	COPD nurse	<1	17
H3^c^	Respiratory consultant	5	18
H4^c^	Respiratory consultant	3	2
H5^d^	Respiratory consultant	6	9
C6^e^	Respiratory service manager	2	25
C7^c^	Lead COPD nurse	2	13
C8	COPD nurse	14	21
C9^c^	Lead physiotherapist	6	10
C10	Assistant practitioner	7	10
C11	COPD nurse	12	12

^a^Identifiers prefixed with H are from the hospital and C are from community care.

^b^COPD: chronic obstructive pulmonary disease.

^c^Study champions were contact points that helped to coordinate research sessions.

^d^H5 was invited to participate in the study by H3 after Respire was designed.

^e^C6 was involved in early discussions but was unavailable to participate in the design process.

### Data Analysis

This qualitative study followed an interpretivist approach that emphasizes the social construction of individuals’ knowledge based on their lived experiences [59]. This approach was appropriate because of the exploratory nature of this study, which focused on HCPs’ experiences providing COPD care. We used 2 techniques for data analysis.

Stages 1 and 2 used content analysis to determine the presence and frequency of specific themes within discussions [60]. Content analysis was selected because of the volume and nature of the data collected. We carefully read the transcripts and assigned codes to references to specific types of data, reasons the data are needed, and comments about data visualization. Stage 3 used inductive thematic analysis to analyze the interview data. We used bottom-up open coding to assign codes to the data at the sentence level based on what the data described. We then grouped those codes to create broader themes that described the entire data set [61]. This analytical approach was chosen given our desire to be more exploratory in stage 3, focusing on capturing the nuances of interacting with Respire.

### Understanding Data Needs (Stage 1)

The first stage involved 5 workshops to explore the data needs for Respire. There were 2 separate workshops with the hospital (H1, H3, and H4) and community HCPs (C7, C8, C9, C10, and C11) each before uniting in the final workshop. Sessions were organized to suit HCPs’ availability and lasted between 30 and 90 minutes in quiet rooms at the clinical sites. Plans for workshops 1 and 2 are provided in Multimedia Appendix 1. We analyzed workshop transcripts after each session.

The first workshop aimed to understand the data needs and develop a shared language between HCPs and researchers. The discussions focused on their patients with COPD and services. We asked, “What data do you want to see about your COPD patients/service?” and “How would you want to interact with that?” In addition to verbal discussions about data needs, to stimulate discussion, HCPs created basic sketches of how data might be visualized. Basic sketching was used as a visual communication tool, enabling researchers to understand HCPs’ mental model of how the data might be tangibly presented. After the sessions, we used the sketches to create wireframes of basic user interfaces and to complement the data analysis. We provided the wireframes in later workshops as stimulus materials.

Subsequent workshops explored the data needs in detail using the wireframes to structure discussions. The HCPs revised the way the data were visualized on the wireframes and refined the included data. For instance, they supplemented the tables with graphs and removed data that they no longer saw as a priority on reflection. We updated the wireframes after the session.

The final workshop gave HCPs from both organizations the opportunity to discuss one another’s wireframes. Two business intelligence staff members, who had been involved in earlier stages of the project [24], attended to share knowledge about the general existence of the included data within both organizations.

### Developing Respire (Stage 2)

**Figure 2:**
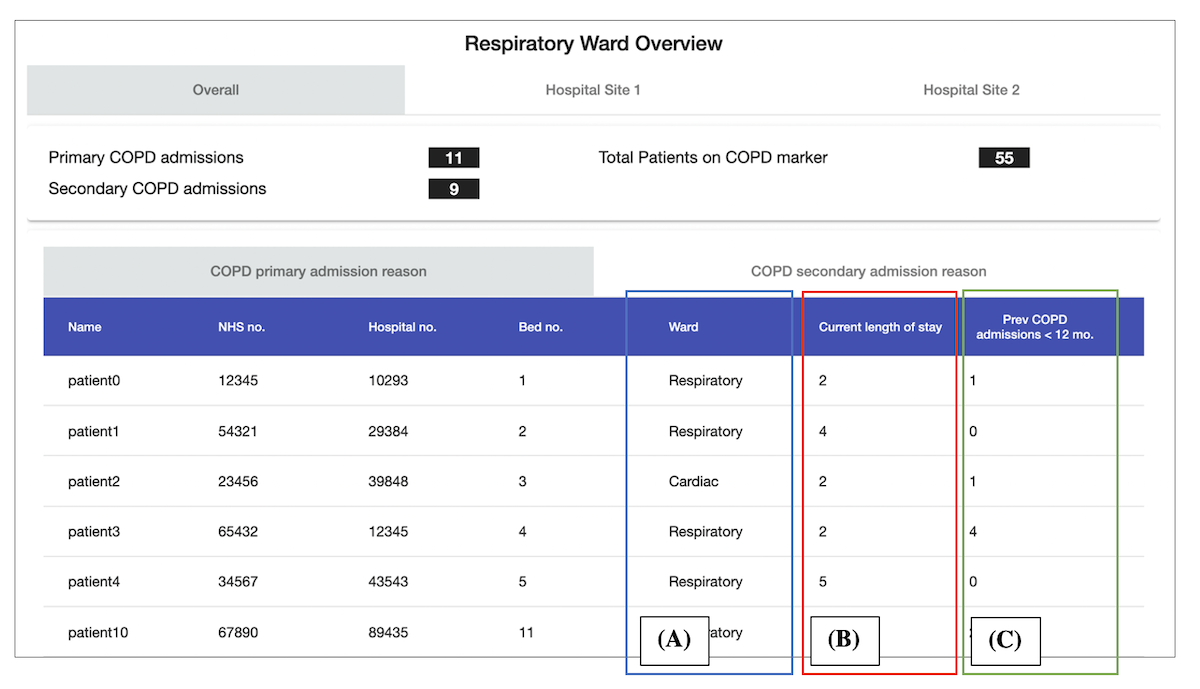
Scenario 1: respiratory ward overview (annotated). This view lists the patients with chronic obstructive pulmonary disease (COPD) in hospital for a COPD-related reason. (A) lists the ward that the patient is on, (B) details each patient's current length of stay in days, (C) details the number of COPD hospital admissions each patient has had in the past 12 months.

**Figure 3:**
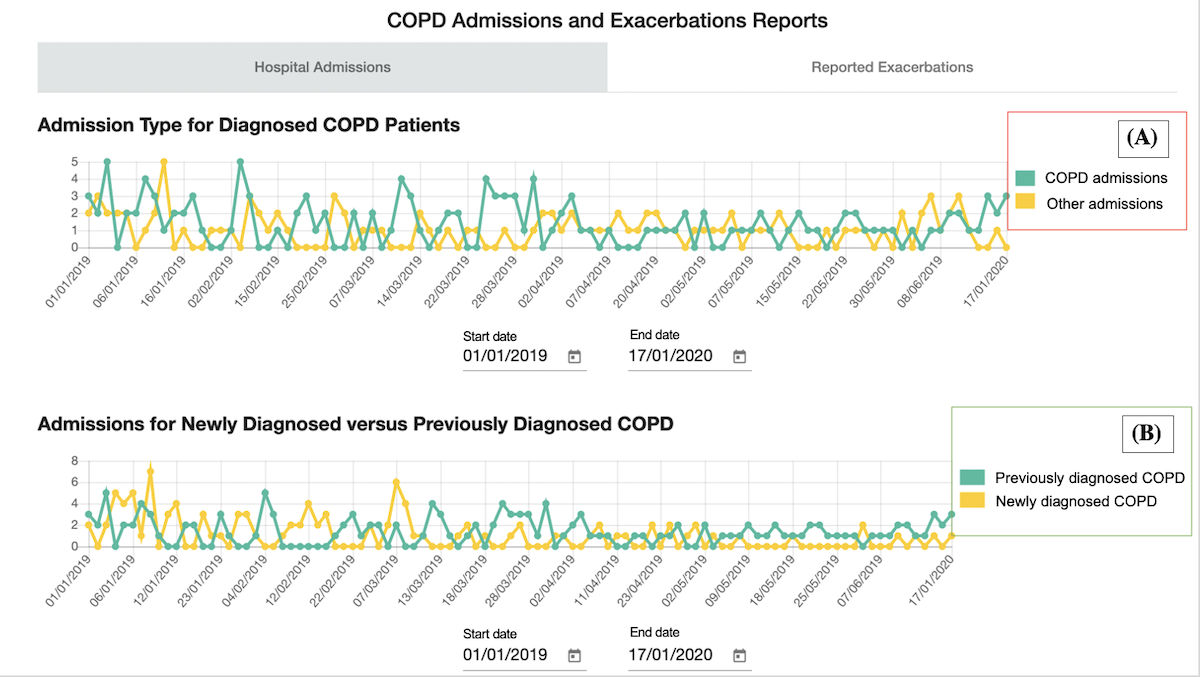
Scenario 2: Admissions and Exacerbation Reports (annotated) showing overall hospital admissions. (A) shows whether the admission was chronic obstructive pulmonary disease (COPD) related or not (B) shows COPD-related hospital admissions split between patients previously known to have COPD and those newly diagnosed because of the admission.

**Figure 4:**
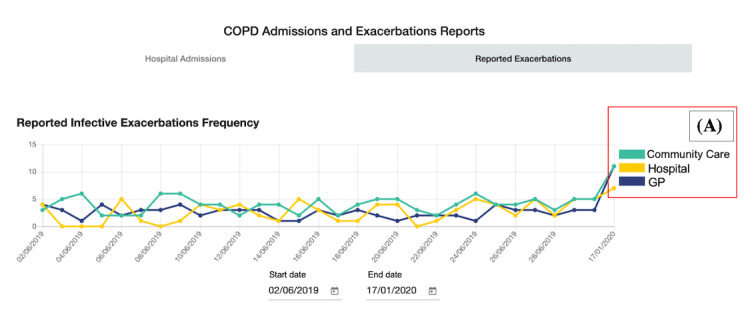
Scenario 2: Admissions and Exacerbation Reports (annotated) with a tab for overall reported infective exacerbations and chronic obstructive pulmonary disease (COPD) hospital admissions. (A) shows which service reported the exacerbation. GP: general practitioner.

**Figure 5:**
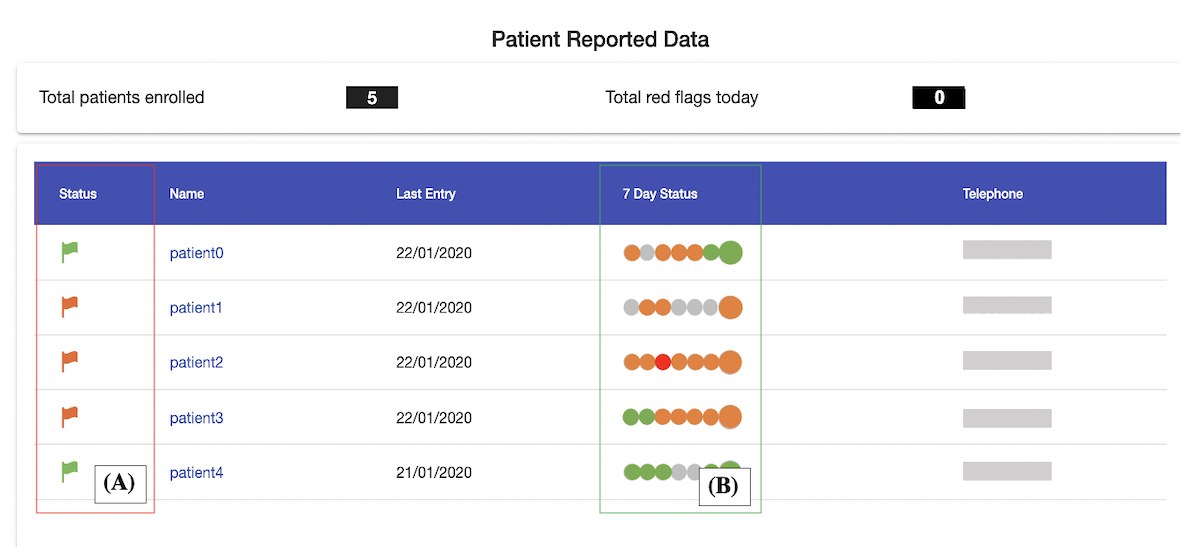
Scenario 3: patient-generated data overview (annotated). (A) Patients’ latest symptom status (green indicates asymptomatic, amber indicates symptomatic, and red indicates severe symptoms); (B) the traffic light system depicting patients’ 7-day status (gray indicates no data have been entered by the patient).

**Figure 6:**
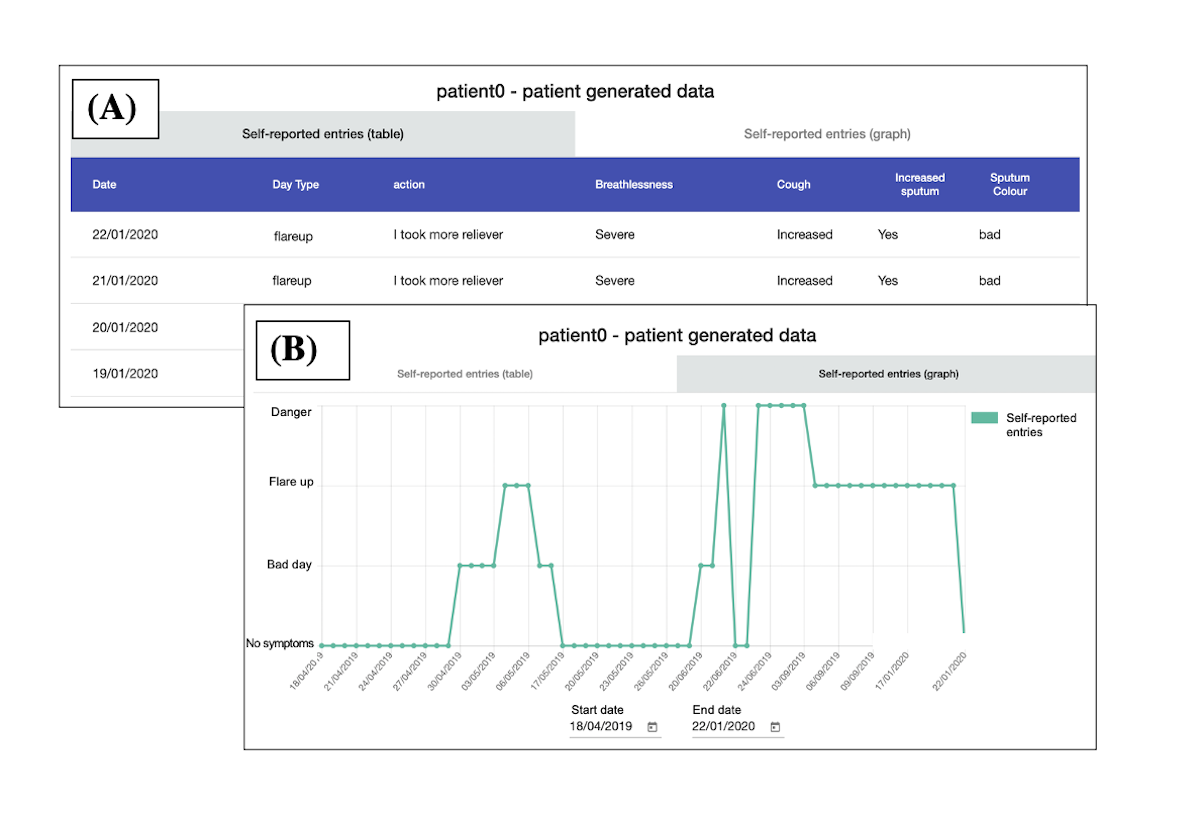
Scenario 3: patient-generated data individual entries (annotated). (A) A log of a patient’s symptom entries and (B) the logged entries in graph format.

**Figure 7:**
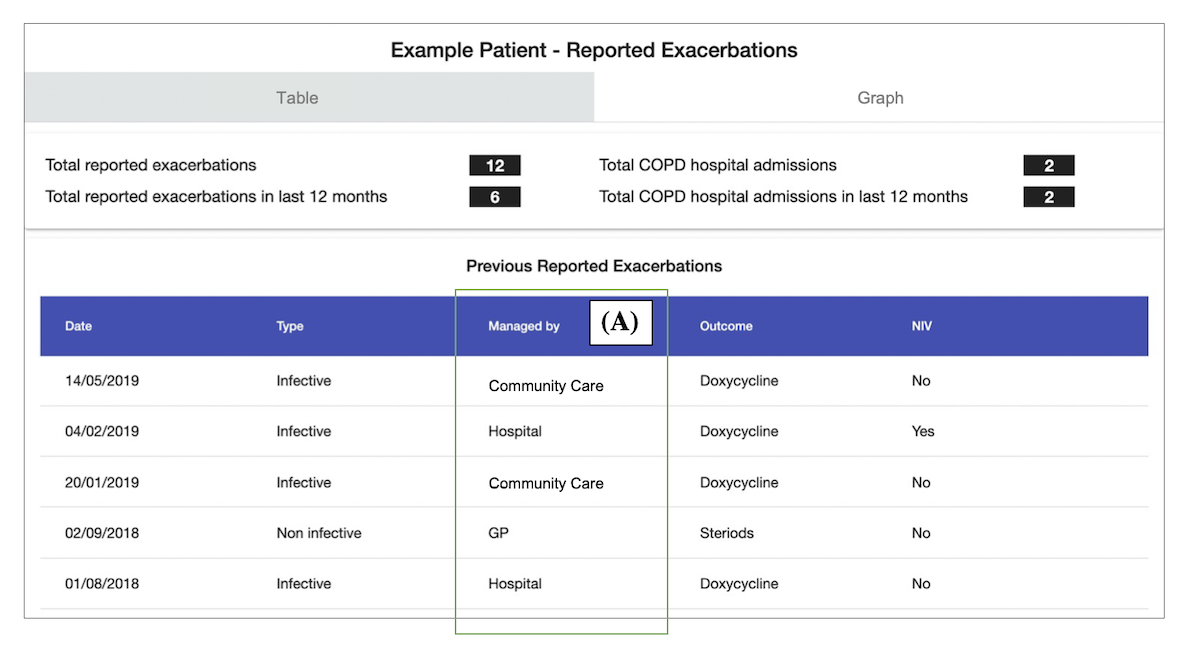
Scenario 4: example patient’s exacerbation history. The table shows a history of a patient’s clinically reported chronic obstructive pulmonary disease (COPD) exacerbations; (A) shows which service has managed each exacerbation. GP: general practitioner.

**Figure 8:**
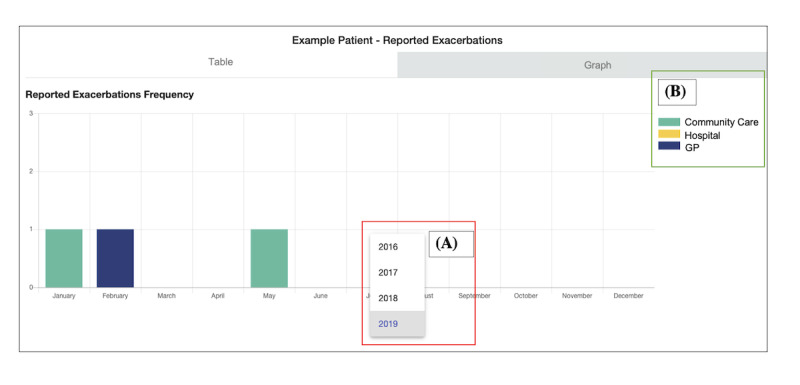
Scenario 4: example patient’s exacerbation history. The graph represents the frequency of the clinically reported exacerbations of a patient with chronic obstructive pulmonary disease over time; (A) shows how health care professionals can filter by year; (B) shows which service has reported the exacerbation. GP: general practitioner.

**Figure 9:**
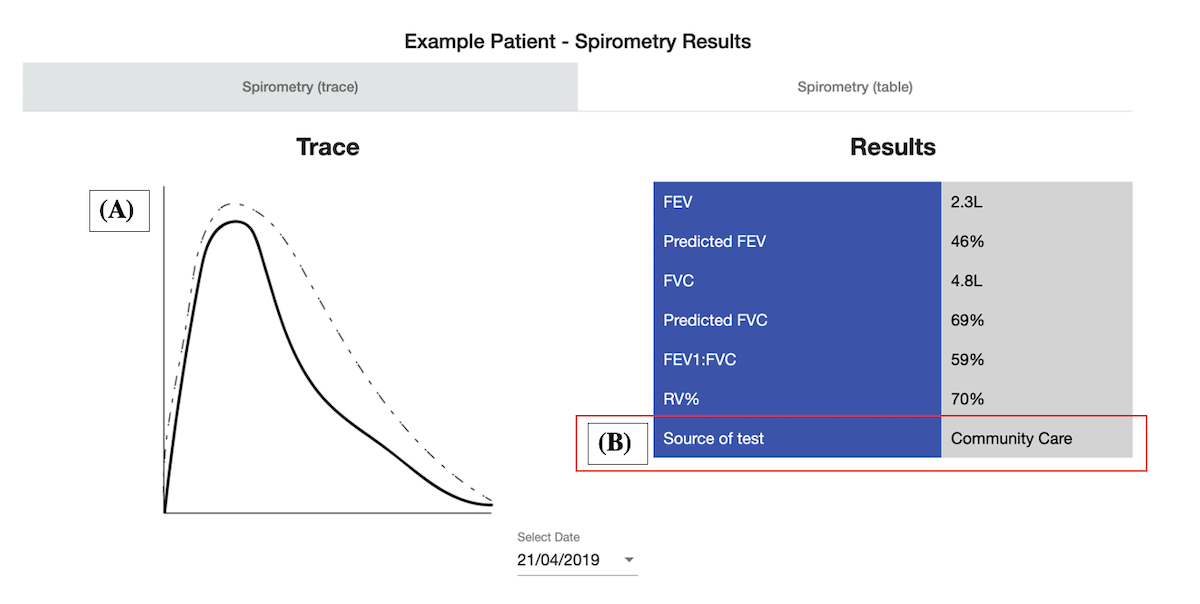
Scenario 5: example patient’s spirometry results; (A) the spirometry trace for the test result; (B) where the test was taken. FEV: forced expiratory volume; FVC: forced vital capacity; RV: residual volume.

**Figure 10:**
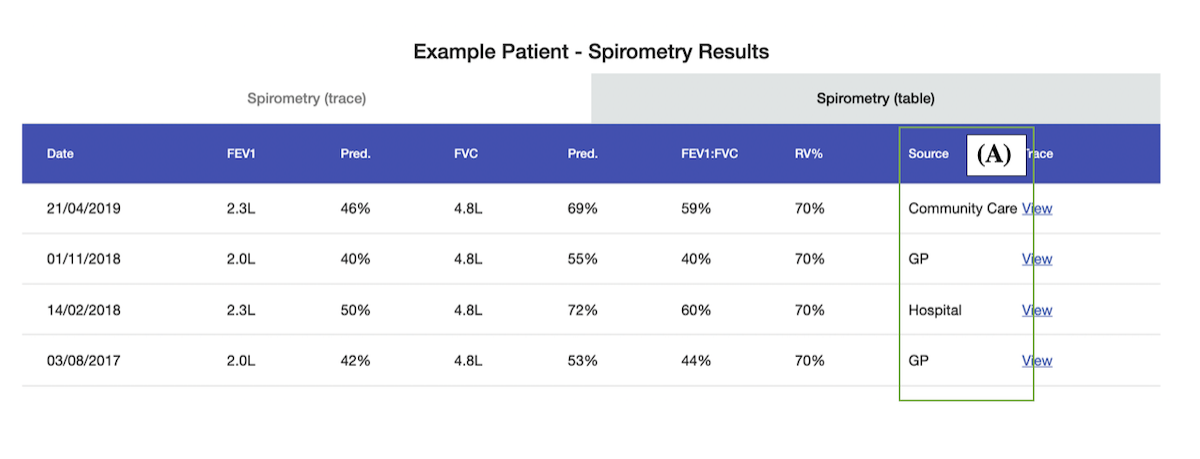
Scenario 5: example patient's spirometry results. The table shows a breakdown of all the spirometry test results for a patient; (A) which service the test was taken at. GP: general practitioner. FEV: forced expiratory volume; FVC: forced vital capacity; RV: residual volume.

We reviewed the data needs captured in stage 1 and chose 5 key use cases to expand into data interaction scenarios for Respire (Figures 2-10). We decided to focus on the needs common to both organizations, as these appeared to be most impactful. For example, we created a scenario based on viewing a patient’s spirometry test result history, as this was an unmet need voiced by both organizations. Spirometry tests require the patient to blow into a device used to diagnose and monitor respiratory conditions. Both organizations discussed these data as being a prominent pain point in practice; thus, it was an important scenario to explore further.

Following this, we confirmed with H4 and C7 if the 5 chosen scenarios (Table 2) were an important focus. We then created Respire, which is an interactive digitization of the 5 data scenarios. Respire is a web app populated with test data to support dynamic interaction. During development, we met with available HCPs (H2, H3, H4, C7, C8, and C9) for feedback on the early versions. For example, ensuring that the wording of headings and the test data made sense for each scenario. Table 2 details the 5 shortlisted scenarios, including a description of the scenario and where the data contained within the scenario would be reported from. A snapshot of each scenario on Respire is shown in Figures 2-10.

**Table 2:** The 5 shortlisted data scenarios.

Number	Scenario name	Scenario description	Data reported by
1	Respiratory Ward Overview	List of in-patients with COPD^a^, the ward they are on, length of stay, and their number of previous COPD-related admissions	Hospital
2	Admissions and Exacerbation Reports	Reports on population-level COPD hospital admissions and exacerbations. Live and historical data can be viewed	Hospital, community care, and GP^b^ practices
3	Patient-Generated Data Overview	View of patients using a mobile app to self-monitor their cough, breathlessness, sputum production and color, and actions in response to symptoms	Patients with COPD
4	Example Patient’s Exacerbation History	Overview of clinically reported exacerbations of a patient with COPD and which service reported them	Hospital, community care, and GP practices
5	Example Patient’s Spirometry Results	A full history of spirometry test results of a patient with COPD and which service the test was taken at	Hospital, community care, and GP practices

^a^COPD: chronic obstructive pulmonary disease.

^b^GP: general practitioner.

### Exploration of Respire (Stage 3)

The final stage explored Respire with 11 HCPs (Table 1). We gathered feedback about their interaction with the scenarios, exploring how they might support their decision-making regarding COPD care. A total of 11 one-to-one sessions were held in quiet rooms at both clinical sites, lasting between 60 and 90 minutes. HCPs were tasked with walking through each scenario and imagining that they had access to it in practice. We asked HCPs to interact with each scenario freely by exploring different tabs, reviewing and interacting with the visualizations, and examining the (test) data. They were asked to think aloud [62]. The semistructured session plan is contained in Multimedia Appendix 2. During the sessions, we asked, “How could the data presented to you in this format influence your decision-making?” “Are there any challenges that you could envisage when using this scenario?” “Who do you think needs to be involved in the collection and maintenance of this data to ensure it is useful?” These questions would provide insight into how HCPs envisage digital tools, such as Respire, might be used in practice.

After reviewing each scenario, HCPs rated (on a 7-point Likert scale, with 1 representing *strongly disagree* and 7 representing *strongly agree*) how realistic they perceived the digital data scenario to be (“the scenario responds in a way that you would expect when using a system to complete this task”) and its relevance to their job (“this scenario is something you would use in your role”). The former was asked to understand if the scenarios were presented realistically to inform them how well they could engage with them and respond to questions. The latter was asked to understand whether there were scenarios that were more relevant to the responsibilities of some HCPs compared with others. Both these responses would add further framing to the discussion.

To conclude the sessions, HCPs ranked the 5 scenarios against each other in order of usefulness (with a score of 1 being the most useful). This would help to discuss the respective strengths and weaknesses of each scenario in context with another.

### Ethical Considerations

This study received ethics approval from both Lancaster University’s Faculty of Science and Technology Ethics Committee and the Health Research Authority (reference: 17/HRA/3092). All participants were required to read an information sheet and sign an informed consent form before participation. All sessions were audio-recorded, with full permission from the participants.

## Results

### Understanding Data Needs (Stage 1)

The data requirements shared by both the hospital and community care were identified from the stage 1 workshop and are summarized in Textbox 1. They largely focused on (1) understanding the severity of a patient’s condition and (2) managing the demands of both health care services. A full list of the data requirements captured from the stage 1 workshop is provided in Multimedia Appendix 3.

**A summary of the hospital and community care’s shared data requirements with direct quotes from participants during stage 1.**

**Patient’s spirometry result history**

- “To *know* if it’s definitely COPD. Then if it is, then what was it [the result] before, does it mean that it’s getting worse [now]?” [H4]
- “The shape of the curve [trace]...will tell you potentially a bit more about their airways. We generally just have the numbers.” [C9]

**Patient’s previous chronic obstructive pulmonary disease (COPD) hospital admissions and exacerbations**

- “When you see patients from admission to admission you might not necessarily join everything together.” [H3]
- “In a certain timeframe how often have they been admitted? Three plus exacerbations, then I would consider that is a suitable patient for us [to manage as opposed to the GP].” [C9]

**Patient-generated data about their COPD symptoms**

- “What has the patient’s perspective been of their illness...we need to understand what the patient understands about their illness.” [H3]
- “Capturing exacerbations and deterioration earlier to avoid potential hospital admissions and potential deterioration.” [C9]

**Patient’s respiratory medications and breathlessness rating**

- “[It] impacts upon how we might manage them...have they been getting more breathless...have their treatments changed?” [H3]
- “If they’d been given a rescue pack of antibiotics and steroids [to take at the onset of exacerbations at home]...[and] to know if they’ve had, say, 6 antibiotics in the last 3 months.” [C8]

**Respiratory interventions a patient has had**

- “[You say] this patient has had 2-3 admissions needing non-invasive ventilation (NIV), have you thought about domiciliary NIV? Or they’ve not done pulmonary rehab in over a year...could you do that?” [H4]
- “You could see what’s been offered or if they’ve been referred [for interventions] and declined.” [C9]

**Live list of COPD-related admissions at the hospital**

- “How many have been there [on the ward] since last week that we need to target first so we can facilitate their discharge?” [H4]
- “[Currently the system] brings up a list of COPD patients...it won’t say whether the particular admission is because of their COPD.” [H3]
- “We actually need to be targeting some of these [admitted] patients that aren’t accessing us [in community care].” [C7]

### Exploration of Respire (Stage 3)

The following sections outline the findings from the stage 3 interviews, including Likert questionnaires, scenario ranking, and qualitative feedback.

#### Quantitative Scenario Feedback

Table 3 presents the results of the Likert questionnaires, showing the mode of participants’ ratings across each scenario and the frequency of the mode. For the scenario realism and relevance scores, 7 indicated *strongly agree*, 4 indicated *neither agree nor disagree*, and 1 indicated *strongly disagree*.

**Table 3:** Results from the stage 3 Likert questionnaires.

Scenario number and scenario			Realism score^a^					Relevance score^b^		
			Mode^c^		Frequency of mode		Mode^c^			Frequency of mode
1	Respiratory Ward Overview	7		7		7			7	
2	Admissions and Exacerbation Reports	7		4		1			4	
3	Patient-Generated Data Overview	7		4		6			5	
4	Example Patient’s Exacerbation History	7		7		7			7	
5	Example Patient’s Spirometry History	7		5		7			7	

^a^“The scenario responds in a way that you would expect when using a system to complete this task.”

^b^"This scenario is something you would use in your role.”

^c^7 indicates *strongly agree*, 4 indicates *neither agree nor disagree*, and 1 represents *strongly disagree*.

The most common realism rating was *strongly agree* (score=7) across all scenarios. Scenarios that commonly received the highest relevance ratings were scenario 1 (Respiratory Ward Overview), scenario 4 (Example Patient’s Exacerbation History), and scenario 5 (Example Patient’s Spirometry History). Scenario 2 was commonly rated the lowest for relevance (Admissions and Exacerbation Reports). Usefulness scores are presented alongside the qualitative findings in the following sections for context.

#### Qualitative Scenario Feedback

##### Scenario 1: Respiratory Ward Overview

Scenario 1 was ranked as the most useful (ranked first place by 6 participants), with the main benefit being that HCPs could quickly identify patients who required support. Hospital HCPs believed that the length-of-stay indicator would help identify patients who have been in hospital the longest to prioritize during ward rounds. It would also help assign senior staff to patients with the longest stays, as these patients are likely to have complex health needs. Similarly, viewing the number of each patient’s previous COPD hospital admissions would allow them to be supported in specific ways. For example, patients without previous admissions may benefit from education on managing their condition. Patients with many previous admissions may require end-of-life care. Community care HCPs felt that the scenario could help identify patients who were in the hospital for their COPD to offer support on discharge. Currently, to achieve this, they must “trawl” [C7] through a list of discharged patients known to have COPD without easily seeing why the patient had been in hospital.

Knowing the data source that would populate scenario 1 was key for HCPs to consider its drawbacks when making their decisions. HCPs explained that the 2 existing data sources that could show which patients with COPD were in hospital had inaccuracies. The first data source was a list of patients with COPD from their data flag system. This system flags patients diagnosed with COPD by local GP practices, hospitals, or community care. However, it is not a “true list” [C7] as (1) patients on the list sometimes “have other respiratory conditions” [C11] and are incorrectly diagnosed with COPD and (2) the system is “not utilized very well” [C7] as flagging patients is a manual process and some patients “probably slip through the net” [C7]. The second data source was the hospital’s clinical coding department. The initial coding of a patient’s hospital admission reason is done by emergency department staff, who are usually “generalists” [C6], and their working diagnosis does not always reflect the final reason for admission. In addition, “very umbrella type codes” [C6] within current classification systems (such as International Classification of Diseases, Tenth Revision [63]) indicate that multiple codes can describe a single hospital admission. For example, COPD may be coded as either *COPD* or *breathlessness*. This means that admissions coded as *breathlessness* could have been missed from the data set that populates scenario 1.

##### Scenario 2: Admissions and Exacerbation Reports

Scenario 2 was ranked the fourth most useful scenario (ranked fourth place by 5 participants), with the main benefit around supporting service planning. For example, to see “where people are referring themselves [when they are unwell]...that first presentation [of symptoms]” [C9], so that the service can identify where they may need extra resources. Forecasting hospital admissions was another way to plan services based on the data as “GP [appointment] spikes normally occur slightly before admission spikes, so if there is starting to be a GP spike then you can follow the trend” [H5]. HCPs discussed how this scenario would be checked on a “monthly” [H4] basis.

However, a key challenge for scenario 2 was the perceived lack of a consistent understanding of COPD exacerbations across hospitals, community care, and GP practices. The HCPs strongly believed this affected the quality and reliability of reporting, as exacerbations are labeled “too easily” [C9]. H4 described this in detail:

It’s easy to label them [patients] as having an exacerbation and give them a little bit of steroids and a little bit of antibiotics...it comes back to how much do you trust the person who is saying they have taken it seriously and taken it to say this is an actual exacerbation?

Which was echoed by C11:

I also do feel like from a professional side that medics are like “well we’ll give you this [treatment for an exacerbation] because it’ll move you on through and out the system”...I do think there’s a bit of discrepancy

about what exacerbations are

The differences in exacerbation reporting were thought to exist because not all HCPs who see patients with COPD are specialists in COPD. The hospital’s and community care’s specialism in COPD makes their identification of exacerbations more reliable, compared with GP practices and emergency department staff who generally do not have COPD “expertise” (H1). Furthermore, HCPs without COPD expertise could assume the patient “knows their condition best” (H1) when approached about a suspected exacerbation and thus provide treatment for an exacerbation.

##### Scenario 3: Patient-Generated Data Overview

**Figure 11:**
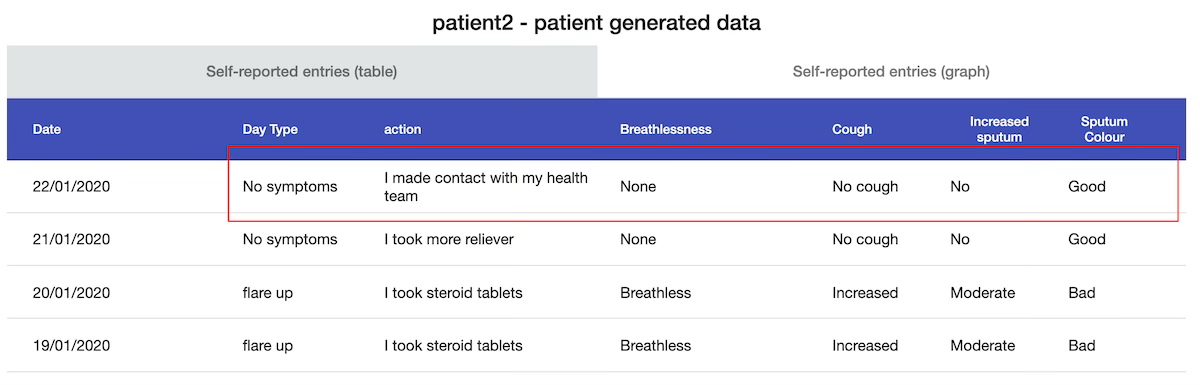
Scenario 3: patient-generated data overview showing an example patient’s symptom log where they had no symptoms but contacted their health care team.

The usefulness ranking for scenario 3 was bimodal, ranked least useful by 3 participants and second most useful by 3 participants. This scenario was seen as valuable for understanding the overall patient experience of living with COPD. HCPs felt that it could be used as a tool to educate patients on their condition. In the clinic, the data could be “an entry to a conversation” [C6] about what actions the patient could take when experiencing certain symptoms. For example, when looking at the (test) data, H4 saw a patient in contact with their health care team despite reporting no symptoms (Figure 11). They felt the patient could have anxiety about their COPD and need “assurance,” with discussions focusing on how the patient could help themselves when they feel anxious. C6 discussed using the data similarly to suggest to the patient “some breathing techniques to help,” so they could distinguish breathlessness caused by anxiety versus an exacerbation. This is important, as anxiety can influence feelings of breathlessness, which patients might not differentiate from a respiratory exacerbation [64-66].

However, identifying the right patient group for self-monitoring is crucial for scenario 3 to be “constructive” [C6]. For patients stable in their management, self-monitoring may be “medicalizing their condition” [C6] and be a reminder “that they are ill” [H5]. However, HCPs felt that acutely ill patients with several previous hospital admissions could benefit from self-monitoring. Newly diagnosed patients could also self-monitor to become familiar with their symptoms. HCPs talked about patients using this platform for a specified period of time for encouragement rather than indefinitely. For example, C9 suggested that patients “could be put on it for a month and monitored by the community care team” to combat 30-day hospital readmissions that occur with COPD [67]. In addition to identifying the right patient group, it was also important for patients to input the required data. Consistent data entry would provide a “true overall reflection” [C10] of a patient’s condition, to be “sure about the day-to-day changes” [C8]. Others felt that sporadic data entries could be acceptable as long as the data are entered when the patient is symptomatic. For example, H5 discussed how:

if you have loads of grey [no input] and then three red [severe symptoms], you know you need to phone them...but there will also be patients who just don’t put data in until they are unwell. What you don’t want is patients who put greens [asymptomatic] but don’t put the red.

Some HCPs discussed how, in certain contexts, asymptomatic days could be “hidden” [C9] from view as “there’s no need to worry about them” [C9]. Despite this, HCPs stressed the importance of recognizing a patient’s symptom-free period, which could be challenging to reinforce if there is a focus on recording only when symptomatic.

Following the need to receive enough data to support decisions, HCPs felt concerned about what missing data might mean and the resources required to investigate. C7 felt missing data could indicate that a patient may “potentially be at home isolated or be dead.” C6 described the likely process of investigating missing data:

You ring them [patient] up and they don’t answer, really common...you maybe try every day for a month. At some point, you are gonna have to send them a letter or do something else” which eventually leads to “generating a whole heap of work...you’ll get through to the patient who will say ‘ah yeah I didn’t bother, I’m not bothered about it anymore’.

There were also concerns about investigating the data that *had* been received. HCPs raised a key issue about feeling responsible for the data that the patient reports. C6 highlighted that remote setups are “implying somebody is monitoring it [the data]” and patients “may become dependent” on the idea that the HCP is continually “looking at that [data] and acting.” However, HCPs lack time and resources to instantly detect health concerns from the data. C6 was further concerned that scenario 3 could encourage patients to take less responsibility for their health concerns as “in reality it means a clinician managing them...they aren’t fully self-managing under this scenario.”

Finally, trust concerns were raised regarding the self-assessed symptom data versus quantitative physiological data. Although self-assessed data were valuable to understand quality of life and patient experience, it was not reliable as “some patients will overreport their symptoms and some will under-report” [H6]. For instance, breathlessness and fatigue have a “huge psychological element” [H2] that can influence how patients perceive their symptom severity. The benefit of physiological data is “you’ve got a guidance that you can say ‘that’s acceptable, that’s not acceptable’” [C8]. However, HCPs felt self-assessed data, paired with physiological readings, were best for identifying what support to offer patients:

if someone was telling me they feel absolutely awful...but actually their physiological parameters were fine, I’d feel more reassured that perhaps they aren’t clinically deteriorating, but obviously I still need to address the fact that the patient feels like they are.

H2

##### Scenario 4: Example Patient’s Exacerbation History

Scenario 4 was ranked jointly as the second most useful scenario (ranked second place by 5 participants), and the main benefit was a better understanding of the patient’s condition journey. More specifically, “how patients’ quality of life and clinical health has been affected across all sectors of care” [H5], as HCPs see patients at specific intervals depending on the concern and “what you don’t see is what’s been happening and how many times” [H5]. This was particularly valuable, as COPD is managed by a diverse clinical team and having *“*the overall picture rather than just snippets of information” [C8] was important for effective care planning. Collating data in this way could also reveal patients who are struggling with their condition and may require a specialist referral or need “advance care planning” [H3]. Overall, HCP felt seeing past exacerbations in this way was an improvement over asking the patient about their history and shuffling through “thousands of records” [C6], with detail that is too “heavy” [H5] and “not relevant for what we [respiratory HCPs] are interested in” [C6].

Scenario 4 could also provide context for the patient’s experience of living with COPD. H4 envisioned using the scenario when engaging with patients in clinics, whereby “you sit with them to say ‘tell me what happened there’” about each exacerbation to learn about their experiences and triggers. This was seen as a valuable communication aid, as patients’ impromptu recall about their experiences “isn’t always great” [H3]. H4 added that better understanding patients’ experiences can support conversations around management:

if they are only breathless because they have seen something on the television that upset them...that has affected the way that they are feeling, but physiologically they don’t need steroids

to manage it

##### Scenario 5: Example Patient’s Spirometry

Scenario 5 was ranked the third most useful scenario (ranked third place by 4 participants). Although it was seen as being “really useful*”* [H5] and time saving, it was perceived as less impactful to patient care than other scenarios. The main benefit was observing how a patient’s lung function may have changed over time. This was possible by comparing the spirometry result history in the table. The trace of each spirometry result alongside its numerical reading was “really important” [C11] for decision-making. This was because the trace helped to determine the “quality” [C9] of the test, it tells HCPs “how the patient performed [during the test]*”* [C11].

However, HCPs highlighted that the trustworthiness of spirometry test results was a key concern. HCPs felt that results from tests taken by hospital HCPs were most reliable, as not all HCPs are adequately trained to deliver spirometry tests effectively. They also felt more confident about tests taken by HCPs or services in which they had a close working relationship. C7 discussed how their close working relationship with the hospital HCPs meant they were aware of each other’s specialisms and competencies in COPD and spirometry. They described how they placed confidence in the test results from the hospital over those from GP practices:

I can see on this one (pointing to spirometry results on the screen) that this was done here [in community care], and this one at the Hospital, so you’d be more inclined to use the Hospital data as kind of reliable, that’s your reliable one, then you can probably work from that as to whether or not the others were really done properly.

C7

In the abovementioned example, the hospital’s result was used as a baseline to judge the reliability of the rest of the results. The HCPs place different “confidence intervals” [H4] on the data, depending on their source. This approach was observed in other HCPs: “was that [spirometry test] actually done by the hospital or community care? In which case, then it’s reliable. Otherwise, it might have been a GP” [C9]; “I definitely believe what came from the hospital over the GPs” [C8]; and “I know you’ve got who’s done the trace, so I think that gives you an idea of the reliability of it” [H5].

## Discussion

### Principal Findings

This study explored how DSDM technology could support COPD care. We achieved this by designing a scenario-based research tool with HCPs to understand human-data interaction for decision-making. DSDM technologies have clear potential to connect HCPs with pertinent data to inform decisions. However, we have unearthed important challenges and lessons relevant to the success of DSDM technologies in practice, which are of particular relevance to the human factors research community: (1) data recorded by HCPs may not be trusted for decision-making, (2) transparency about data sources is required to trust and understand data, (3) sporadic and subjective data generated by patients have value but create challenges for decision-making, and (4) HCPs require support to interpret and respond to new data and its use cases.

### Data Recorded by HCPs May Not Be Trusted

Data were considered most trustworthy when the HCP who recorded it was perceived as an expert in assessing COPD. Previous work has shown that the source of medical information determines its adequacy for use in decision-making [68-70]. Specifically, Cicourel [68] observed how the perceived credibility of medical information was based on social and professional hierarchies. For example, they found that diagnostic information from attending physicians was rarely challenged and perceived as more objective than that of medical students [68]. In our study, data recorded by the respiratory ward staff at the hospital (perceived as highly specialized in COPD) were considered the most trustworthy, whereas data generated by GPs (perceived as less specialized in COPD) were considered the least trustworthy.

It was easier to assess if the data were trustworthy when it was produced by a familiar colleague, enabling the HCP to assess the colleague’s skills and competencies. Jirotka et al [71] described this as “biographical familiarity,” a predicate for trust. They observed how mammogram readers became familiar with the strengths and weaknesses of their colleagues, affecting how they read the mammograms produced by different centers [71]. Similarly, our study shows how a lack of biographical familiarity impacts HCPs’ engagement with data from staff with unfamiliar competencies. In contrast, the hospital and community care trusted each other’s data, as they were familiar with one another’s competencies.

Awareness of how trust impacts the use of data across departments and organizations is important and impacts how data should be displayed on DSDM technologies. Respire showed the *source* of spirometry test results and exacerbation reports, which HCPs felt were crucial contextual metadata to emphasize. One possible way to support building trust with unfamiliar data could be through *seals of approval* or *digital badges* built into dashboard designs [72]. For example, a badge representing skill proficiency could be displayed next to entries from organizations that have had specific training in spirometry.

### Transparency About Unreliable Data Sources Is Needed

Knowing which data sources were populating Respire was important for assessing any limitations when using data to make decisions. The explicit mention of a system’s data sources is also important for building trust [73]. This emphasizes the need to make data sources transparent to users, addressing the human-data interaction challenges of legibility [46]. Certain data sources were perceived as unreliable, such as data from the coding department and the hospital data flag system. The unreliability of coded data has been explored in previous work [74-76], particularly the overlap of codes for a single clinical condition [74]. As the specificity of medical data is tailored to the original purpose of its collection, repurposing it requires additional details for the data to be usable in new contexts [77,78].

We argue that transparency about the data sources that populate DSDM technologies will enable HCPs to assess important contextual factors about the data. This supports the use of data in new contexts. Transparency can be achieved by labeling the data sources on the user interface and visually representing their reliability. For example, data from the coding department could have icons alongside it, which indicate that the code is a working diagnosis or overlaps with other respiratory conditions.

### Subjective Data Recorded by Patients Is Challenging for Decision-making

Despite the benefits of viewing patient-generated data, self-assessed data may be too variable for decision-making. Previous work has shown that it is challenging for patients with COPD to answer subjective questions about breathlessness and coughing [79]. To address this, patients may underreport symptoms unless there are large deviations from their baseline [79]. Unreliable reporting of symptoms impacts how data are interpreted by HCPs, which means that patients may not receive the care they require. This concern relates to the human-data interaction challenge of legibility, making data transparent and comprehensible [46].

We found that there are contexts in which subjectivity in patient-generated data is acceptable, such as clinical discussions about perceived symptoms and quality of life. Scenario 3 presented symptom data in a structured format, enabling HCPs to quickly pinpoint moments in time. Patient-generated data, in turn, becomes a useful resource for HCPs and patients to collaboratively identify personalized management strategies and goals [80]. Therefore, although quantitative symptom readings can address variability in patient reports [81], complete quantification of a patient’s chronic health experience removes an important perspective. A combination of quantitative and subjective data can provide a holistic view of a patient’s condition to support the development of personalized goals. However, patients may require support to understand their data in preparation for clinic visits to maximize the value of the co-interpretation process [80].

To support patients’ understanding of their data, digital technologies for self-monitoring could prompt them to input written context alongside symptom changes; for example, if symptoms deviate from a baseline. The written context prepares patients to discuss key moments in the clinic visit. However, in contexts where HCPs receive data remotely, HCPs may feel concerned if they deem themselves liable to address the content of patients’ free-text notes outside of clinic visits [82]. To mitigate this, when viewing patient-generated data remotely, typed notes could be inaccessible until the HCP interacts with the patient directly. Future work is needed to explore how to connect context to symptoms without causing these concerns in HCPs.

### Sporadic Data Entry by Patients Has Value

A notable challenge with patient-generated data is the perceived effort required to encourage patients to record data consistently so that health patterns can be identified [83,84]. Thus, sporadic data entry can mean that important insights are missed or rendered ambiguous [85]. For instance, sporadic data can cause challenges where complete data are required to predict exacerbations [86]. Similarly, sporadic data may indicate that a patient is too unwell to monitor their symptoms [85,87,88]. However, we found that sporadic data could have value depending on the use case.

Patients’ symptomatic days were key information for HCPs, as this required some action from them. Therefore, HCPs suggested that recording data about being unwell would be a valuable insight, despite the absence of recording asymptomatic days. Patients who prefer to reduce their time thinking about their condition may also prefer to record data only when symptomatic [10]. HCPs suggested that Respire could have a filter that only displayed patients who were symptomatic and required support. However, when applying filters to data sets, HCPs may inadvertently pay less attention to patients outside the filtered subset [27]. Therefore, filters applied to the views of patient-generated data should have alerts regularly reminding the user of the applied filter.

It should be highlighted that enabling patients and HCPs to discuss health improvements is important [89,90]. Therefore, we do not argue that asymptomatic days should not be tracked as they can provide a measure, and a reminder, of how frequently patients feel *well*. Rather, we have found in contexts where there is no hard requirement to record data each day, a focus on symptomatic days alone can provide value. Future work should further explore such use cases to identify the key opportunities for sporadic data.

### Support and Clear Processes Are Needed When Interacting With New Data

Concerns about responding to data can impact HCPs’ desire to integrate data into their workflow [88,91-93]. This relates to the human-data interaction challenge of agency, regarding acting on data and its implications [46]. Patient-generated data present a novel opportunity to support decision-making. However, HCPs were concerned that they would be expected to instantly investigate (lack of) data and the work involved in meeting this expectation. They felt that patients may stop acting on their health concerns as they expected HCPs to closely monitor them. In addition, HCPs had no guidance on the investigation and interpretation of patient-generated data. Aligning expectations about responding to data is important for HCPs to use data in practice [88,93]. Bardram and Frost [88] observed similar challenges raised by nurses that were responding to low mood reported by patients with bipolar disorder. This challenge highlights how wider sociotechnical considerations influence how HCPs engage with data.

Future studies should explore aligning expectations and establishing processes for responding to patient-generated data to alleviate concerns. This can be achieved by working with HCPs to understand the patient segments [94] who they wish to receive data from, the data required, and the frequency of its collection. Following this, we can collaboratively design appropriate workflows, dataflows, and digital interfaces. Our study found 4 use cases for patient-generated data: (1) supporting discussions in clinic visits, (2) monitoring acute patients to detect deterioration, (3) temporary monitoring of patients discharged from the hospital, and (4) temporary monitoring of newly diagnosed patients. Each use case may benefit from different processes, data, and visualizations. Balancing HCPs’ data needs with patients’ expectations in different contexts can support an understanding of how these systems can work in practice.

### Limitations

This study has 2 important methodological limitations. First, the exploration of Respire consisted of the same HCPs who informed its design (except for H5 and C6). Involving the same HCPs in the design could have introduced a positive bias into the feedback, with participants potentially responding more positively to Respire [95]. The second limitation is that this research was undertaken with 2 National Health Service organizations in North West England. Their local context and ways of working have shaped our findings, which require acknowledgment when transferring the findings to other health care contexts [50].

### Conclusions

By exploring data interaction scenarios with HCPs, we unearthed lessons and design implications for DSDM technologies in the context of COPD care. Although DSDM technologies can support HCPs, there are important human-data interaction and sociotechnical challenges that influence their design and deployment. These challenges are related to (1) trusting data for clinical decision-making, (2) navigating unreliable and incomplete data sets, and (3) interpreting and responding to new types of data. Further investigation of these challenges will enhance the design and deployment of effective DSDM technologies for health care. Although COPD was our area of focus, we argue that our findings have the potential to translate [50] to other areas where DSDM technologies might be used in health care.

## Appendix

Multimedia Appendix 1 Plan for data needs workshops.

Multimedia Appendix 2 Plan to explore Respire.

Multimedia Appendix 3 Stage 1 data requirements.

## Abbreviations

COPD: chronic obstructive pulmonary disease
DSDM: data-supported decision-making
GP: general practitioner
HCP: health care professional
REST: Representational State Transfer
