# Supplementary material for: Exploring Human-Data Interaction in Clinical Decision-making Using Scenarios: Co-design Study
Source: JMIR Hum Factors. 2022 May 6;9(2):e32456. doi: 10.2196/32456 (PMC9123541; doi:10.2196/32456)
Supplement: Multimedia Appendix 1 [file humanfactors_v9i2e32456_app1.docx]

# Multimedia Appendix

This is a Multimedia Appendix to a full manuscript published in the J Med Internet Res. For full copyright and citation information see http://dx.doi.org/10.2196/jmir.xxxx

The following sections present the workshop plan for the stage one methods.

# Understanding Data Needs: Session 1 Plan

**Aim:**

- Learn more about the Hospital and Community Care HCPs data needs and how these may be visually presented to them for interaction

**Materials:**

- A3 paper
- Pencils and erasers
- Wireframes (if applicable)
- Dictation device
- Spare consent forms

**Structure:**

| **Activity** | **Time** |
| --- | --- |
| - Introduce the workshop and its aims and objectives.   - Reassure participants that they do not need to be artists to sketch of ideas and concepts. - Introduce research team’s role and brief participants about discussing complex topics with non-clinical research team. - Seek consent to start audio recording. | 10 minutes |
| - Discussion of data needs prompted by key questions. - Aim to split first half of session focusing on data needs on a patient level and second half on wider service level data needs. - Start by asking the wider group to each think about the following questions:   - What data would you want to see about your COPD patients and service?   - What pain points are you experiencing in your role relating to data?   - *Suggest participants to use the materials provided to write or sketch ideas as a means to communicate / express their points.* - Ask each participant to share what they’ve focused on and then ask the following, encouraging the rest of the group to join the discussion:   - What made you choose to focus on this?   - How would you want to interact with that data?   - What impact would visualising this data have on your role?     - What impact would that have on patient care?   - Do you currently have access to this data?     - How / why not?     - What system does it sit in?     - How is it recorded?     - How is it visualised / accessed? - If participants do not use any materials provided, researchers can roughly sketch discussed ideas and present them back for comment to stimulate discussion.   - “Do you mean like this?”   - “Does this capture what you’re describing?”     - “Could you show me what you mean?” | 30 mins |
| - Summarise the data needs discussed throughout the session   - Briefly go over each topic area discussed | 5 mins |
| - Debrief participant and discuss next steps   - Will take discussion points / sketches and create some stimulus to provide back in the next session.   - Questions from participants | 5 mins |

# Understanding Data Needs: Session 2 Plan

**Aim:**

- Present wireframes created from the previous workshop and take a deeper dive into the data needs discussed and their visualisation

**Materials:**

- Wireframes printed on A3 paper
- Pens and pencils
- A3 paper
- Dictation device

**Structure:**

| **Activity** | **Time** |
| --- | --- |
| - Introduce the workshop and its aims and objectives. - Refresh participants’ memory of previous session. - Introduce the wireframes, explain what they are, and how they were created.   - Highlight that these are not ‘final designs’ for an interface but are used to stimulate discussion. The aim is to update and refine them iteratively. Feel free to critique / change / amend what is shown.   - “Feel free to annotate the wireframes using the pens provided” - Seek consent to start audio recording. | 10 minutes |
| - Present first wireframe to participants and refresh their memory about the discussions that led to its creation.   - “Does this capture what we discussed in the last session?”   - “Would this data alleviate the pain points you raised in the previous session?”     - Why / why not   - “What would you change about it?”   - “How might you interact with this?” - Repeat for remaining wireframes. | 30 mins |
| - Summarise the data needs discussed in detail during the session and changes to the wireframes. | 5 mins |
| - Debrief participant and discuss next steps   - Wireframes will be amended based on discussion in the session   - Next steps would be to unite both organisations to compare and discuss data needs and wireframes     - Ask participants who else should attend   - Questions from participants | 5 mins |
