# Supplementary material for: Exploring Human-Data Interaction in Clinical Decision-making Using Scenarios: Co-design Study
Source: JMIR Hum Factors. 2022 May 6;9(2):e32456. doi: 10.2196/32456 (PMC9123541; doi:10.2196/32456)
Supplement: Multimedia Appendix 2 [file humanfactors_v9i2e32456_app2.docx]

# Multimedia Appendix

This is a Multimedia Appendix to a full manuscript published in the J Med Internet Res. For full copyright and citation information see http://dx.doi.org/10.2196/jmir.xxxx

The following sections present the semi-structured interview plan for the stage three methods.

# Exploring Respire: Session Plan

**Aim:**

- 1:1 semi-structured sessions with HCPs to walk through each scenario on Respire and explore their interaction and how the scenarios could support them with decision-making about COPD care.

**Materials:**

- Laptop to access Respire (web app)
  - Dongle (back up internet)
- Questionnaires (printed)
- Dictation device

**Structure:**

| **Activity** | **~Time** |
| --- | --- |
| - Introduce the interactive web tool and refresh participants’ memory of how the research team got to this stage based on previous activities with the HCPs. - Explain Respire is a prototype, e.g., not a real or complete system, uses test data, will require some envisioning. | 5 minutes |
| - Introduce aims and objectives of the session.   - Seek consent to start audio recording | 3 minutes |
| - Participant to walk through each of the five scenarios from 1 to 5 (~10 minutes per scenario) - Researcher to ask questions about each following a semi-structured approach.   - Ask participant to rate the realism and relevance of each scenario after they have finished interacting with it | 40-50 mins |
| - Once walkthrough is complete ask participant to rank each scenario in order of its usefulness.   - Discuss participant’s choices and compare first and last | 5 mins |
| - Debrief participant - Time for participant’s questions | 3 mins |

**Key questions when exploring each scenario:**

Exploration of each scenario will be conducted using a semi-structured approach, with core questions listed below for each scenario.

*Scenario 1: Respiratory Ward Overview*

1. How could the data presented to you in this format influence your decision-making?
2. How do you envisage using this scenario in your day to day work?
3. Are there any difficulties you might have engaging with this kind of data as it is presented to you?
4. Does this scenario provide you will all the information that you need to identify which patients have come in with a COPD related exacerbation?
5. Are there any challenges that you could envisage when engaging with this scenario in practice?
   1. How could these be alleviated?
6. Who do you think needs to be involved in the collection and maintenance of this data to ensure it is useful?
7. Is this scenario easy to navigate and understand?

- ***Hand the participant the questionnaires***

*Scenario 2: Admissions and Exacerbation Reports*

1. How could the data presented to you in this format influence your decision-making?
2. How do you envisage using this scenario in your day to day work?
3. Are there any difficulties you might have engaging with this kind of data as it is presented to you?
4. Who do you think needs to be involved in the collection and maintenance of this data to ensure it is useful?
5. Do you know how this data is currently reported?
6. Is this scenario easy to navigate and understand?
7. Do you know how Community Care/GP practices/the hospital currently report this data?
8. Are there any challenges that you could envisage when engaging with this scenario in practice?
   1. How could these be alleviated?

- ***Hand the participant the questionnaires***

*Scenario 3: Patient Generated Data Overview*

1. How could the data presented to you in this format influence your decision-making?
2. How do you envisage using this scenario in your day to day work?
3. Are there any difficulties you might have engaging with this kind of data as it is presented to you?
4. To what extent could this scenario support your current and future work?
5. Who do you think would need to be involved in the collection of this data to maintain its usefulness?
6. Is this scenario easy to navigate and understand?
7. How might you act upon this data as it is presented here?
8. Are there any challenges that you could envisage when engaging with this scenario in practice?
   1. How could these be alleviated?

- ***Hand the participant the questionnaires***

*Scenario 4: Example Patient’s Exacerbation History*

1. How could the data presented to you in this format influence your decision-making?
2. How do you envisage using this scenario in your day to day work?
3. Are there any difficulties you might have engaging with this kind of data as it is presented to you?
4. Who do you think would need to be involved in the collection of this data to maintain its usefulness?
5. Is this scenario easy to navigate and understand?
6. How might you act upon this data as it is presented here?
7. Are there any challenges that you could envisage when engaging with this scenario in practice?
   1. How could these be alleviated?

- ***Hand the participant the questionnaires***

*Scenario 5: Example Patient’s Spirometry Results*

1. How could the data presented to you in this format influence your decision-making?
2. How do you envisage using this scenario in your day to day work?
3. Are there any difficulties you might have engaging with this kind of data as it is presented to you?
4. Who do you think would need to be involved in the collection of this data to maintain its usefulness?
5. How might you act upon this data as it is presented here?
6. Is this scenario easy to navigate and understand?
7. Are there any challenges that you could envisage when engaging with this scenario in practice?
   1. How could these be alleviated?

- ***Hand the participant the questionnaires***

***Ask the participant to rank the usefulness of each scenario***
