# Supplementary material for: Exploring Human-Data Interaction in Clinical Decision-making Using Scenarios: Co-design Study
Source: JMIR Hum Factors. 2022 May 6;9(2):e32456. doi: 10.2196/32456 (PMC9123541; doi:10.2196/32456)
Supplement: Multimedia Appendix 3 [file humanfactors_v9i2e32456_app3.docx]

# Multimedia Appendix

This is a Multimedia Appendix to a full manuscript published in the J Med Internet Res. For full copyright and citation information see http://dx.doi.org/10.2196/jmir.xxxx

The following sections provide an overview of the data requirements discussed in the stage one workshops. Requirements centered on understanding the severity of each patient’s condition and the demands on both healthcare services.

## Hospital Data Requirements

The Hospital HCPs discussed nine data requirements, described below:

1. Access to the number of previous COPD exacerbations and admissions that a patient has had including “*whether they needed steroids, antibiotics, nebulizers*” (H1) to understand their severity, as “*when you see patients from admission to admission you might not necessarily join everything together*” (H3).
2. Seeing a history of the patients’ spirometry test results (including the trace and the numerical result) taken at the Hospital, Community Care and GP practices, so that the HCPs “*know if it’s definite COPD? Then if it is, then what was it [the result] before, does it mean that it’s getting worse?”* (H4), as they do not always get access to these results.
3. A view of the current COPD patients admitted onto the respiratory ward in the Hospital, including “*how many are new admissions and how many have been there since last week that we need to target them first so that we can facilitate their discharge*” (H4).
4. Patient-generated data about quality of life and symptoms to identify “*how many patients that we can detect from that data are having a worsening in their symptoms currently in the community to allow us to be able to go in and target those early and prevent an exacerbation or admission*” (H3) and could support self-management.
5. View a summary about each patients’ medication list, comorbidities, history of their breathlessness score, and if they have a Do Not Resuscitate form as these factors “*impacts upon how we might manage them*” (H3).
6. View live data on exacerbations being managed by Community Care to understand demand, including “*having a map of the area where the hot spots [of exacerbations] currently are, and the staffing ratio in those areas*” so that “*you could see at a glance where the problems are in terms of staffing*” (H3).
7. Access to any past respiratory interventions that a patient may have had and when those were so they can make suggestions for next steps, such as *“this patient has had two to three admissions needing non-invasive ventilation (NIV), have you thought about domiciliary and NIV? Or they’ve not done pulmonary rehabilitation in over a year or over a few years, could you do that?”* (H4).
8. Insight into the prevalence of influenza in the local community, such as positive influenza tests or COPD patients being treated with flu medication though they did not “*know if it’s possible or not … [or] know if this data is even collected*” (H3), as “*in flu season we see a huge surge in admissions and exacerbation”* (H3).
9. Insight into environmental data though “*we don’t really know how well this relates to exacerbations terrible well*” (H3) but “*some patients will be atopic”* (H3) to pollen and pollution, which could link to worsening of symptoms.

## Community Care Data Requirements

Community Care HCPs discussed nine data requirements, described below:

1. Seeing a history of the patients’ spirometry test results to see *“if they’ve been accurately diagnosed with spirometry”* (C11) and *“where have they had the spirometry?”* (C9), including the *“the shape of the curve [trace] … it will tell you potentially a bit more about their airways. We generally just have the numbers … you look at it together”* (C9).
2. View a patient’s history of COPD specific hospital admissions and exacerbations to identify *“in a certain time frame how often have they [the patient] been admitted”* (C9) and their *“length of stay”* (C7) to understand how a patient is managing their COPD, as if they’ve had *“three plus exacerbations then I would consider that is a suitable patient for us [to manage as opposed to the GP]”* (C9).
3. View patient-generated data about COPD symptoms for “*capturing exacerbations and deterioration earlier to avoid potential hospital admissions and potential deterioration*” (C7) and to support conversations with patients “*if a person who’s on the app rings in then we can go back and look at that [data]”* (C9).
4. See the respiratory interventions a patient has had, such as their “*most recent pulmonary rehabilitation attendance*” (C9), “*smoking cessation*” (C7), “*home support episodes”* (C9), and *“patients that have declined it [those services]”* (C8) to understand which interventions to offer to patients and the uptake of these services.
5. View number of diagnostic and annual spirometry tests taken at GP practices as it helps to understand “*how much is being carried out in GP practices rather than in our service [which also provide spirometry]*” (C8) to oversee service utilisation and demand for spirometry tests.
6. View service level data about pulmonary rehabilitation referral and completion rates, clinic referral rates, and home support referral rates for reporting and to understand where *“we have been really busy”* (C9).
7. View an overview about individual patients including “*up to date medication, respiratory medication*” (C9) such as *“an antibiotic and steroid profile”* (C7), *“smoking status*” (C9), annual “*dyspnoea scores and COPD Assessment Tests*” (C7) and “*when inhaler techniques have been checked”* (C9).
8. View the number of COPD related hospital admissions from patients on Community Care’s caseload *“over the past year”* (C7) as it would be beneficial to see *“how effective the services are”* (C9), such as “*to compare our episodes of [patients needing support while on] home support with [hospital] admissions*” (C9) and see *“patients with the most hospital admissions” (C9)* in case “*we actually need to be targeting some of these patients that aren’t accessing us [Community Care’s services]”* (C7).
9. An overview of the COPD patients reaching end of life, including how often each patient has received face to face or telephone contact and “*“know how many have flared up or how many are just ticking on nicely”* (C11) to ensure care quality guidelines are met as “*if we deemed a patient palliative … we contact them regularly*” (C7)
